# Short-Term Biological Toxicity Prediction of [^177^Lu]Lutetium-Oxodotreotide: An Original Retrospective Analysis

**DOI:** 10.1089/cbr.2023.0195

**Published:** 2024-06-26

**Authors:** Julien Dubois, Guillaume Tosato, Philippe Garrigue, David Taieb, Benjamin Guillet, Vincent Nail

**Affiliations:** ^1^ Department of Radiopharmacy, La Timone University Hospital, CERIMED, Aix-Marseille University, Marseille, France.; ^2^ Department of Radiopharmacy, Montpellier University Hospital, Montpellier University, Montpellier, France.; ^3^ Cancer Research Institute of Montpellier (IRCM), University of Montpellier, Montpellier, France.; ^4^ Montpellier University Hospital, Montpellier University, Montpellier, France.; ^5^ Department of Nuclear Medicine, La Timone University Hospital, CERIMED, Aix-Marseille University, Marseille, France.

**Keywords:** [^177^Lu]Lu-oxodotreotide, peptide receptor radionuclide therapy, radiopharmaceutical, toxicity prediction

## Abstract

**Introduction:**

[^177^Lu]Lutetium (Lu)-oxodotreotide is a radiopharmaceutical drug used as peptide receptor radionuclide therapy (PRRT) for somatostatin receptor–expressing neuroendocrine neoplasms. It provides an additional effective alternative treatment for these rare cancers. Although well tolerated, its safety profile must continue to be characterized to support its use as a first-line treatment or for additional cycles. This study evaluated factors associated with the occurrence of [^177^Lu]Lu-oxodotreotide induced short-term toxicity.

**Materials and Methods:**

A retrospective observational monocentric study was carried out from July 2013 to October 2021. Inclusion criteria were defined as follows: patients who received at least four cycles of [^177^Lu]Lu-oxodotreotide and were followed up for 6 months after the last injection. Graduated toxicity was defined using the National Cancer Institute Common Terminology Criteria for Adverse Events 5.0. Cox regression was used in the analysis.

**Results:**

Forty patients were included. The most frequent toxicities occurred during the first cycle and were graded as G1 or G2. As expected, toxicities were predominantly hematological and hepatic, with incomplete reversibility after each cycle. The following factors were significantly related to the occurrence of hematological or hepatic toxicity during PRRT: gastrointestinal primary tumor diagnosis, bone metastases, peritoneal metastases, pancreatic metastases or pulmonary metastases, and high tumor grade.

**Conclusion:**

Knowledge and consideration of these factors in adjusting [^177^Lu]Lu-oxodotreotide treatment regimen could help prevent or reduce the severity of these toxicities. Further studies are still warranted to refine these results and improve treatment management.

## Introduction

Neuroendocrine neoplasms (NENs) are rare and heterogeneous tumors in their localization and differentiation grade.^1^ The majority of differentiated NENs overexpress somatostatin receptors and particularly the subtype 2 (SSTR2).^2^ The contribution of nuclear imaging in the assessment of NENs is essential for the initial workup and the follow-up after surgery or targeted treatments. The evaluation of SSTR2 overexpression correlates positively with the existence and localization of the primary tumor and distant metastases.^3^ Peptide receptor radionuclide therapy (PRRT) with [^177^Lu]Lutetium (Lu)-oxodotreotide (Lutathera^®^; Advanced Accelerators Applications AAA, Novartis) is indicated for the treatment of inoperable or metastatic, progressive, well-differentiated gastroenteropancreatic neuroendocrine tumors expressing SSTR2 in adults. The recommended therapeutic scheme consists of four infusions of 7,400 MBq of [^177^Lu]Lu-oxodotreotide; distant from 8 weeks. Indeed, [^177^Lu]Lu-oxodotreotide administration is generally well tolerated, otherwise the occurrence of adverse events (AEs) may lead to treatment adaptations, such as delaying the treatment infusion up to 16 weeks, decreasing the dose to 3,700 MBq, or discontinuing the treatment.^4^ These AEs are mostly short-term and resolutive, in relation to hematological toxicities, and appear to be associate with different factors such as patient basal pathophysiologic condition, pathology grades, and cumulative PRRT doses.^5–7^ The identification of factors inducing AEs could allow to anticipate their occurrence and adapt the treatment management. This study aims to identify epidemiological, clinical, and biological factors associated with the occurrence of diverse biological toxicities during treatment with [^177^Lu]Lu-oxodotreotide in a cohort of 40 patients.

## Materials and Methods

### Patients

This study was conducted in accordance with the ethical standards as laid down in the 1964 Declaration of Helsinki and its later amendments or comparable ethical standards. The need for ethics approval was waived due to the retrospective and observational nature of the study (MR-004 French research type). All participants in this study have given written consent to have their data analyzed and published.

**FIG. 1.**
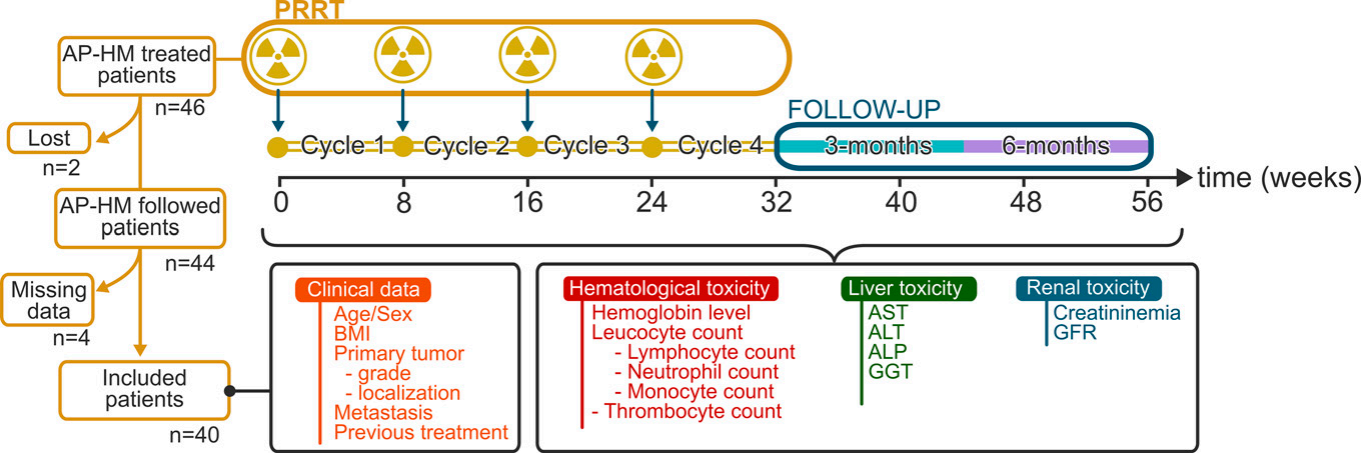
Schematic flow chart of the retrospective trial inclusion. ALT, alanine aminotransferase; ALP, alkaline phosphatase; AP-HM, assistance publique des hôpitaux de Marseille; AST, aspartate aminotransferase; BMI, body mass index; GGT, γ-glutamyl transferase; GFR, glomerular filtration rate; PRRT, peptide receptor radionuclide therapy.

A single-center retrospective observational study was conducted in the nuclear medicine department of the Timone hospital (Assistance Publique des Hôpitaux de Marseille; AP-HM) from July 2013 to October 2021. Forty-six patients were treated with [^177^Lu]Lu-oxodotreotide after a positive single-photon emission computed tomography (SPECT) imaging or positron emission tomography (PET) imaging, respectively, using [^111^In]In-pentetreotide or [^68^Ga]Ga-edotreotide. The patients included in this study were followed for 6 months after receiving at least four cycles of [^177^Lu]Lu-oxodotreotide. Those with insufficient clinical or biological data collection or those lost to follow-up during the inclusion period were excluded (Fig. 1). The administration of [^177^Lu]Lu-oxodotreotide and subsequent monitoring conformed to the instructions in the summary product characteristics (SmPC).^4^ [^177^Lu]Lu-oxodotreotide was administered by slow intravenous gravity injection coadministered with an amino acid solution, Primene^®^ (Baxter), or Lysine–Arginine solution (LysArg) Lysakare^®^ (AAA, Novartis).

### Data management

All data were collected as described by Good Clinical Practices (GCP) guidelines.^8^ Epidemiological, clinical, paraclinical, pharmaceutical, and radiopharmaceutical dispensing data were obtained from patient records and anonymized. The primary tumor localizations were retrieved, and three comparable groups were created: gastrointestinal tract, pancreas, and miscellaneous (meningioma, pheochromocytoma, paraganglioma, and pulmonary) NENs. This stratification has led to the formation of three comparable-sized groups, and allowed us the comparison of the off-label use of [^177^Lu]Lu-oxodotreotide. The pancreas NENs group was also created to meet with the OCCLURANDOM phase II trial (NCT02230176), which was still active during the period of data collection.^9^ Biological assessments were obtained before PRRT initiation, at each participating therapy, every 2 weeks in between, and 3–6 months thereafter. Blood levels for hemoglobin, white blood cell counts (WBC)-neutrophils, lymphocytes, monocytes, platelet counts, creatinine, glomerular filtration rate (GFR), and liver parameters including transaminases (alanine aminotransferase [ALT] and aspartate aminotransferase [AST]), alkaline phosphatase (ALP), and gamma-glutamyl transferase (GGT), were collected. Comparisons were performed on data collected before, during, and after PRRT to determine hematological, liver, and kidney toxic effects.

Biological toxicity grades were defined according to the National Cancer Institute Common Terminology Criteria for Adverse Events (NCI CTCAE) version 5.0.^10^ Toxicities were staged based on their severity, from the lowest grade 1 (G1) to the highest grade 4 (G4). Studied parameter grades for the study are described in Supplementary Table S1.

### Statistical analysis and parameters

Univariate Cox regression was applied to obtain AE predictors. Our relative reference date was the date of first infusion of [^177^Lu]Lu-oxodotreotide. The occurrence of toxicities was treated as a censored event for use in Cox regression models. When the threshold for each considered parameter was reached, the event of toxicity was recognized. The time between the date of the blood analysis and the date of the toxicity was used as censored time. The Wald test was used for the regression parameters. The hypotheses of log-linearity and proportional hazard ratio were then checked for quantitative covariates. The survival and survminer packages were used for Cox analysis using R 4.1.3. Comparisons between AE occurrences were done using unilateral Fisher’s exact test. Statistical significance was defined as *p* ≤ 0.05.

## Results

### Patients

Forty-six patients were treated with [^177^Lu]Lu-oxodotreotide. Six patients were excluded due to lost to follow-up or missing data. A total of 40 patients were included in the analysis [median age: 65 years old; interquartile range (IQR): 55 to 71 years old], composed of 23 males (57.5%) and 17 females (42.5%). The tumor grade was defined for 36 patients (90.0%). Metastases were identified in 36 patients (90.0%) including 23 patients (64.0%) suffering from multiple metastases localizations. Metastatic sites classified as “other” (i.e., pancreatic, lung, parotid glands, and ovarian) were found in 9 cases (22.5%). Prior to PRRT, patients had received one to multiple lines of various treatment regimens in different sequences. This included surgical resection followed by at least one antihormonal treatment, i.e., somatostatin analogues based on recommendations and off-label use at the time. Other treatments comprised targeted therapy, including everolimus or sunitinib; chemotherapy by 5-fluorouracil, in various combinations; chemoembolization; and external radiotherapy (summarized in Table 1). Characteristics of [^177^Lu]Lu-oxodotreotide administration are described in Supplementary Table S2.

**Table 1.** Basic Patient Characteristics, Pathology Characteristics and Therapies Administered Before PRRT Initiation (*n* = 40)

Parameters	Value
Age at first cycle (years), median [IQR]	65 [55–71]
Gender, n_Male_/n_Female_ (sex-ratio)	23/17 (1.4)
Weight (kg), median [IQR]	71 [61–80]
BMI (kg/m^2^), median [IQR]	25 [24–29]
Comorbidities, *n* (%)	26 (65,0%)
*Cardiovascular*	20 (50,0%)
Primary tumor localization, *n* (%)	
*Gastrointestinal tract (small intestine, duodenum, jejunum, stomach)*	18 (45,0%)
*Pancreas*	12 (30,0%)
*Other: meningioma (5), pheochromocytoma (2), paraganglioma (2), pulmonary (1)*	10 (25,0%)
Tumor grade (Ki-67 index ; Mitotic index), n (%)	
*Grade 1 (<3%; 2–20 mitosis/mm^2^)*	11 (27.5%)
*Grade 2 (3–20%; >20 mitosis/mm^2^)*	18 (45,0%)
*Grade 3 (>20%; >20 mitosis/mm^2^)*	7 (17.5%)
*No information*	4 (10,0%)
Metastasis, *n* (%)	35 (87,5%)
*Liver*	28 (70,0%)
*Bone*	12 (30,0%)
*Lymph node*	17 (42.5%)
*Peritoneal*	6 (15,0%)
*Other: pancreatic (3), pulmonary (4), parotid (1), ovarian (1)*	9 (22.5%)
Previous surgical resection, *n* (%)	30 (75,0%)
Previous targeted therapy, *n* (%)	
*Somatostatin analog (octreotide, lanreotide)*	28 (70,0%)
*Everolimus*	15 (37.5%)
*Sunitinib*	8 (20,0%)
*Bevacizumab or pembrolizumab*	3 (7.5%)
Previous systemic chemotherapy, *n* (%)	16 (40,0%)
*5-Fluorouracil*	15 (37.5%)
*Dacarbazine*	11 (27.5%)
*Streptozocin*	6 (15,0%)
*Doxorubicin*	5 (12.5%)
*Platinum compounds (oxaliplatin or cisplatin)*	6 (15,0%)
Previous chemoembolization, *n* (%)	12 (30,0%)
Previous external radiotherapy, *n* (%)	12 (30,0%)

BMI, body mass index; IQR, interquartile range; PRRT, peptide receptor radionuclide therapy.

Nine patients benefited from dose reductions or cycle spacing due to AEs during PRRT. About half of the patients who underwent dose reduction or cycle spacing did so due to a decrease in their platelet counts, while the other half did so due to a decrease their hemoglobin levels.

### Toxicity description

**FIG. 2.**
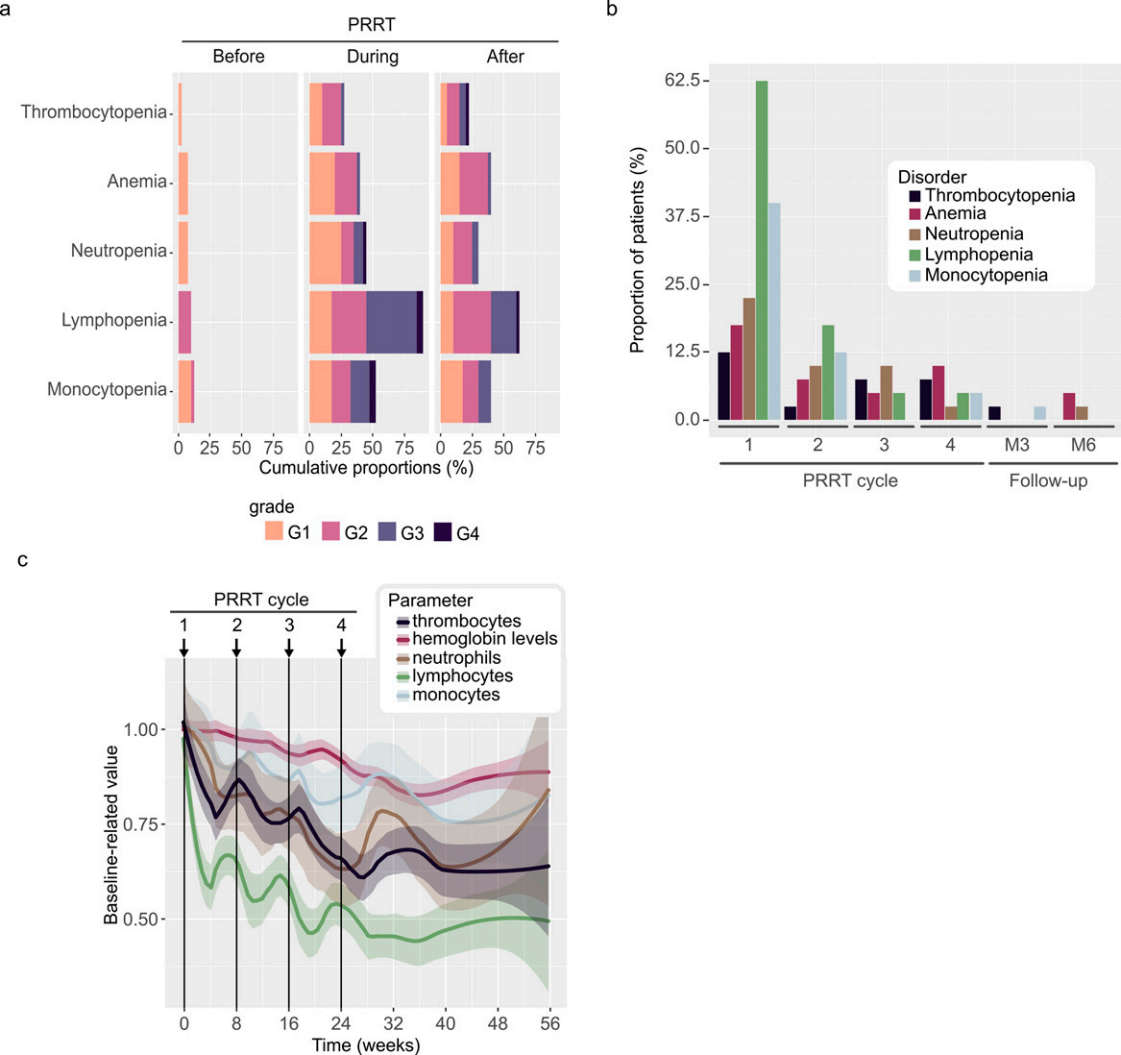
Hematological toxicity, **(a)** Hematological toxicities that have occurred before PRRT initiation, during and after PRRT overall cycles, **(b)** Proportions of hematological toxicity at each PRRT cycle overall grades, and **(c)** Baseline-related evolution of hematological parameters during PRRT (*n* = 40). G, grade; PRRT, peptide receptor radionuclide therapy.

Before PRRT initiation, 11 patients (27.5%) had G1/G2 hematological disorders, with 5 (12.5%) having two blood cell lines impaired. During PRRT, across all cycles, WBC lines were the most affected hematological line. Among them, lymphopenia was the most predominant AE (36 patients; 90.0%), followed by monocytopenia (24; 60.0%) and neutropenia (19; 47.5%). Anemia was observed in 18 patients (45.0%) and thrombocytopenia in 13 patients (33.0%) (Fig. 2a, Supplementary Table S3a and S3b). The highest hematological toxicity rates, across all grades, were observed after the first cycle: lymphopenia (25; 62.5%), monocytopenia (16; 40.0%), neutropenia (9; 22.5%), anemia (7; 17.5%), and thrombocytopenia (5; 12.5%) (Fig. 2b, Supplementary Table S3b). Across all cycles, 14 patients had grade 3 or higher impairment in a single hematological parameter; 3 patients with two hematological parameters; 4 patients with three hematological parameters; and 2 patients with four hematological parameters. A mean relative decrease was observed across all cycles, up to 37% for lymphocytes, 20% for thrombocytes and neutrophils, 10% for monocytes, and 6% for hemoglobin levels. Lymphocyte and thrombocyte counts decreased the most in the first cycle (35% and 16%, respectively) and gradually declined as did the other blood cell lineages throughout the cycles. This decrease became more pronounced after the third cycle for neutrophils and monocytes (10% and 6%, respectively). Nevertheless, after 6 months’ follow-up, no hematological line had recovered its baseline levels (Fig. 2c).

**FIG. 3.**
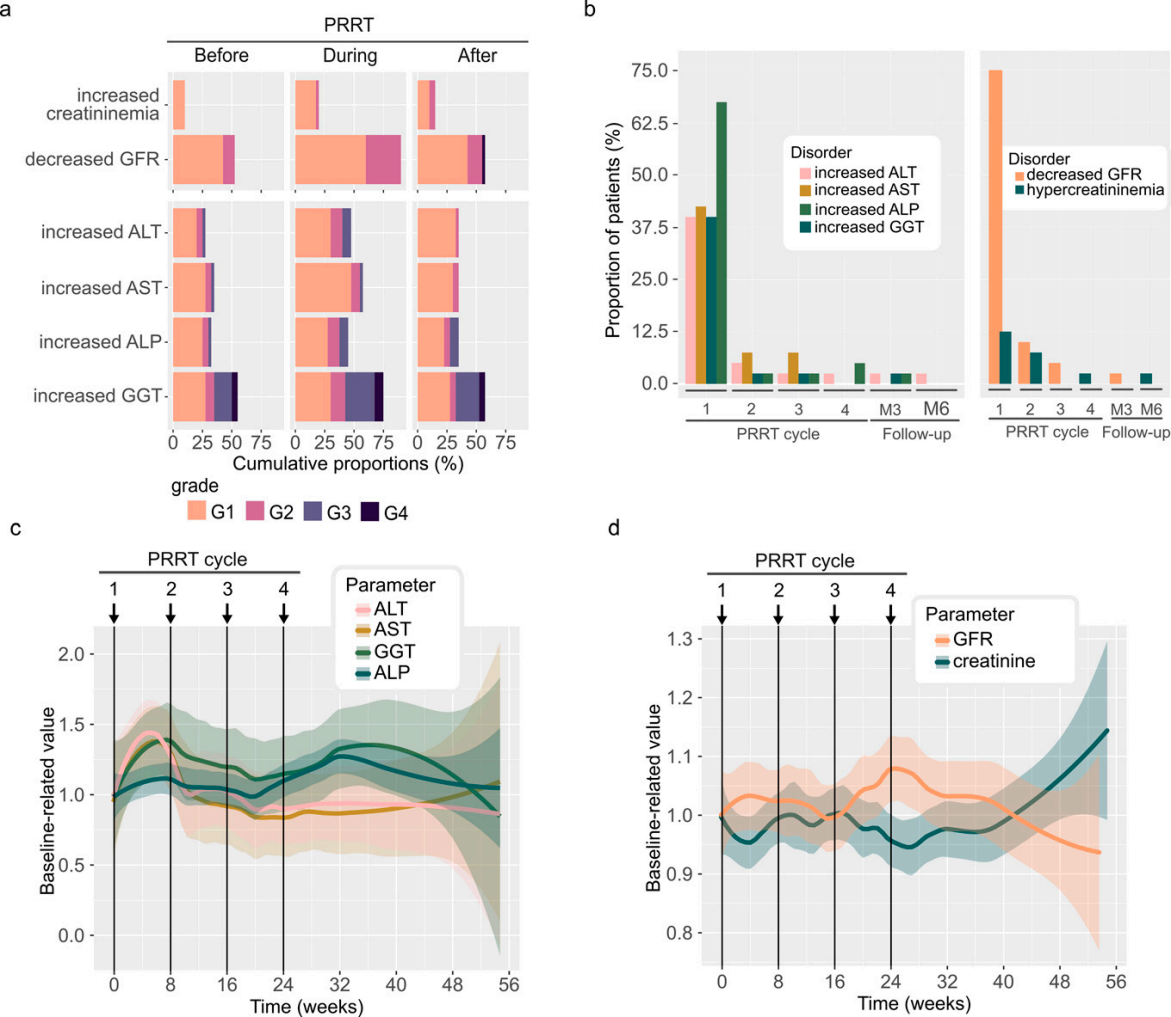
Hepatic and renal toxicity, **(a)** Hepatic and renal toxicities that have occurred before PRRT initiation, during and after PRRT overall cycles, **(b)** Proportions of hepatic (left) and renal (right) toxicity at each PRRT cycle overall grades, **(c)** Baseline-related evolution of hepatic parameters during PRRT, and **(d)** Baseline-related evolution of renal parameters during PRRT (*n* = 40). ALP, alkaline phosphatase; ALT, alanine aminotransferase; AST, aspartate aminotransferase; G, grade; GFR, glomerular filtration rate; GGT, gamma-glutamyl transferase; PRRT, peptide receptor radionuclide therapy.

At the baseline, biological hepatic cytotoxicity (defined by increased AST and ALT levels) and biological cholestasis (defined by increased ALP and GGT levels) were observed in 4 patients (10.0%); 2 patients (5.0%) had biological hepatic cytolysis, 7 patients (17.5%) had biological cholestasis, and 8 patients (20.0%) had isolated GGT increase. During PRRT, various hepatic toxicities were observed in 12 patients (30.0%): 5 patients (12.5%) had G1 hepatic cytolysis, including associated G1 cholestasis in 2 patients (5.0%); 3 patients (7.5%) had G2-associated hepatic cytolysis and cholestasis; and 3 patients (7.5%) had isolated G2 cholestasis. One G3 hepatic cytolysis associated with G2 cholestasis was observed after the first treatment cycle, but not in subsequent treatment cycles. No patient developed liver failure before, during treatment, or 6 months after (Supplementary Table S4a and S4b). The highest hepatic toxicity rates, across all grades, were observed after the first cycle (Fig. 3b, Supplementary Table S4b). Indeed, hepatic biomarkers had the highest mean relative increase after the first cycle, up to 39%, 31%, 34%, and 8% for ALT, AST, GGT, and ALP, respectively, but tended to recover throughout the cycles. After the 6-month follow-up, the hepatic biomarkers stabilized around the baseline levels (Fig. 3c).

Renal toxicity was evaluated through changes in GFR. At baseline, G1 GFR decrease was observed in 18 patients (45.0%), and G2 GFR decrease in 4 patients (10%). During PRRT, 12 patients (30.0%) had G2 GFR decrease. The highest renal toxicity rates, across all grades, were observed after the first cycle, principally because of G1 GFR decrease (Fig. 3a and b, Supplementary Table S4a and S4b). The mean relative variation of creatinine and GFR remained stable, up to 10%, during the treatment and the 6-month follow-up period (Fig. 3d).

### Analysis of the toxicity-related factors

**FIG. 4.**
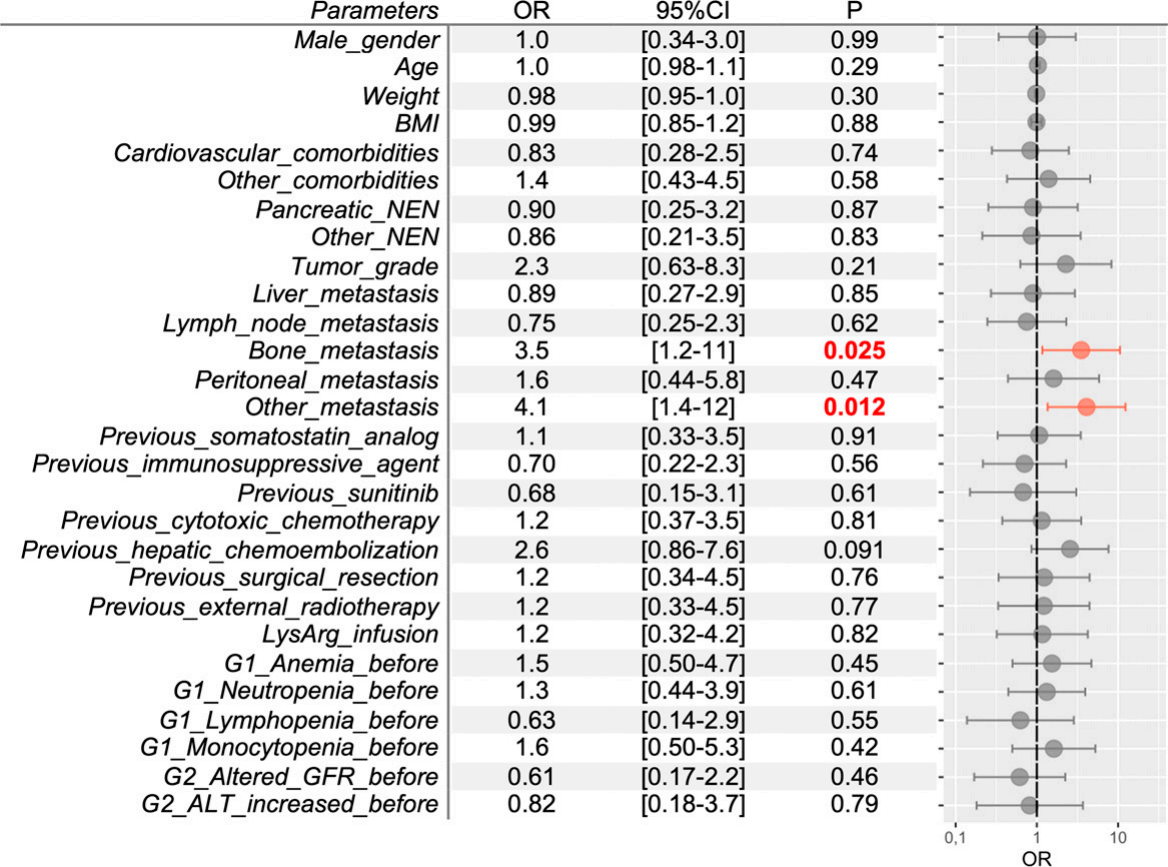
Factors associated with grade 1 thrombocytopenia (<100,000/mm^3^). ALT, alanine aminotransferase; BMI, body mass index; CI, confidence interval; G, grade; GFR, glomerular filtration rate; LysArg, lysine-arginine solution; NEN, neuroendocrine neoplasm; OR, odds ratio.

Bone metastases and other metastases (i.e., pancreatic, pulmonary, parotid, and ovarian) were significantly associated with the occurrence of G1 thrombocytopenia (OR 3.5, *p* = 0.025, and OR 4.1, *p* = 0.012, respectively, Fig. 4). Other factors were found to be nonsignificant. The presence of bone metastases also appeared to be significantly associated with the occurrence of G2 thrombocytopenia (OR 3.3, *p* = 0.047). Two additional factors were found to be associated with G2 thrombocytopenia: having G1 thrombocytopenia and G1 neutropenia (OR 14, *p* < 0.001, and OR 4.7, *p* = 0.049, respectively, Supplementary Fig. S1).

**FIG. 5.**
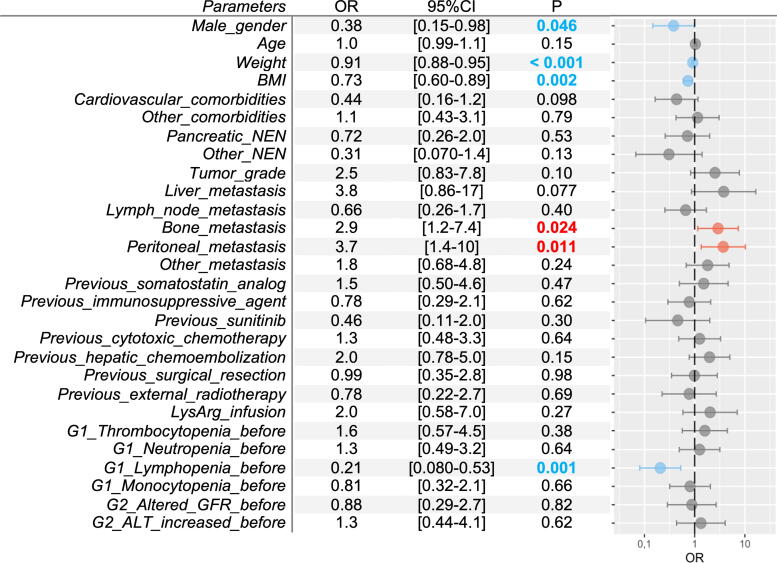
Factors associated with grade 1 anemia (<11.5 g/dL). ALT, alanine aminotransferase; BMI, body mass index; CI, confidence interval; G, grade; GFR, glomerular filtration rate; LysArg, lysine-arginine solution; NEN, neuroendocrine neoplasm; OR, odds ratio.

Bone metastases and peritoneal metastases were identified as G1 anemia–associated risk factors (OR 2.9, *p* = 0.024, and OR 3.7, *p* = 0.011, respectively). Male gender, weight, and body mass index (BMI) were found to be protective factors (OR 0.38, *p* = 0.046, OR 0.90, *p* < 0.001, OR 0.73, *p* = 0.002 respectively, Fig. 5). Weight and BMI were found to be associated G2 with anemia protective factors (OR 0.93, *p* = 0.003, OR 0.72, *p* = 0.008, respectively), whereas peritoneal metastases and previous G1 anemia were identified as associated risk factors (OR 4.4, *p* = 0.023, and OR 9.3, *p* = 0.004, respectively, Supplementary Fig. S2).

Analysis of the factors associated with leukocyte toxicity and hepatic toxicity is detailed in Supplementary Figure S3–S6. Finally, we did not find in our univariate analyses any factor associated with the occurrence of kidney failure for all grades.

## Discussion

The study’s population was comparable with that described in the literature in terms of age, primary and metastatic localization, previous cancer treatment, and occurrence of toxicity.^7,11–13^ The overall [^177^Lu]Lu-oxodotreotide treatment tolerance was maintained for at least 6 months after the last cycle, despite the occurrence of toxicities.^14^ The mean kinetics after each cycle showed a global decrease in hematological parameters and a global increase in hepatic parameters with an altered peak after the first cycle. The mean kinetics of renal parameters showed no alterations up to 6 months. All patients presented an alteration of their basal hematological, liver and/or renal biological parameters, in various grades along with the treatment regimen.

Presence of bone metastases was identified as a significant risk factor for the occurrence of thrombocytopenia, anemia, and monocytopenia. Lutetium-177-based PRRT has enough linear energy transfer to impact bone marrow, for which this toxicity has already been observed with [^177^Lu]Lu-oxodotreotide or [^177^Lu]Lu-PSMA-617.^15–17^ Patients with less frequent metastases, including pancreatic and pulmonary ones, were related to a significantly faster occurrence of thrombocytopenia. The presence of peritoneal metastases was found to be a risk factor for faster anemia onset. While the lesions caused by beta radiation appear to be associated with PRRT, the different toxicities may be related to tumor extensiveness, or both. We also observed that the occurrence of G1 or higher thrombocytopenia or anemia is related to a faster decrease in these cell lines to a higher grade. Better monitoring of such patients is therefore important given the possibility of subsequent fast worsening to G2 or higher AEs. Interestingly, male gender emerged as a protective factor against G1 anemia. This result can be explained by the gender-related variability in the normal value of hemoglobin levels. Gender is no longer a significant factor at a G2 and higher-grade anemia, where standardized thresholds apply equally across genders. Higher weight and BMI were found to be protective factors, which can be explained by the standard dose used with no weight adjustment. Conversely, patients with a higher BMI are at a higher risk of developing anemia.^18^ Tumor grades (≥G2) and gastrointestinal origin were predictors of rapid anemia onset, suggesting that the hematotoxic effects of [^177^Lu]Lu-oxodotreotide might exacerbate underlying conditions such as iron deficiency, vitamin B12 deficiency, or gastrointestinal bleeding.^19,20^ For the three leukocyte lineages, the factors associated with the AE occurrence differed, and their clinical interpretation was difficult. Lymphocytes are known to be more radiosensitive than monocytes, which is confirmed in our study (90% lymphopenia versus 60% monocytopenia, *p* = 0.002).^21^ Patients with previous hepatic chemoembolization appear to be at a heightened risk of fast and severe lymphopenia, which thus should be further monitored. The frequent drop in leukocyte counts should lead to patient monitoring because of the increased susceptibility to infections. We do not advocate for the necessity of anti-infective prophylaxis or hematopoietic growth factor treatment unless there is an occurrence of G4 toxicity.

Factors related to G2 hepatic toxicity could be identified in our study, which could be further explored by including more patients. The presence of peritoneal metastases was a risk factor for a relevant increase in AST and ALT as hepatic cytolysis biological markers. Surprisingly, the presence of hepatic metastases is not associated with an increased risk of hepatic toxicity. This result may be related to the large number of patients with these metastases or related to pre-PRRT hepatic alterations. Patients previously treated by hepatic chemoembolization seem to be predisposed to a faster cholestasis onset. However, there was no significant increase in bilirubin blood levels in this patient cohort. None of the patients developed liver failure, which is supported by a recent retrospective study.^22^ We will not discuss the increased GGT levels risk factors because of their low specificity and ubiquitous nature. Finally, the present study did not find factors associated with any of the considered renal biomarkers because of a low occurrence of patients with renal alterations during [^177^Lu]Lu-oxodotreotide treatment. Before PRRT, 45% had G1 and 10% had G2 decreased renal function. Renal toxicities appeared to be reversible to the basal value except for 1 patient 6 months after the last cycle (G2 GFR decreased). The imputability of PRRT was ruled out for G4 kidney failure appeared in 1 patient 6 months after the last cycle, considered acute and associated with dehydration.

Several patient parameters included in the present study were not relevant for PRRT toxicity analysis, such as age and previous everolimus and/or sunitinib treatment, which were not related to toxicity, as shown previously, despite known hematotoxicity of sunitinib.^23,24^ Also, previous conventional chemotherapy, or external radiotherapy, was not found to be significantly associated with the occurrence of toxicity. The variety of treatment regimens reflects the complex and individualized management approach for this patient population. To the best of our knowledge, no other treatment was introduced before or during the [^177^Lu]Lu-oxodotreotide cycles, except for antiemetics or analgesics. Their imputability on the toxicity occurrences was considered unlikely. Although the type of coadministered amino acid did not significantly alter biological tolerance, we expected it to benefit renal tolerance.^25^ The patient tolerance of any pre-PRRT therapy was not found in their medical records and could therefore not be included in the studied factors. The volume and quantity of metastases were not considered, despite their potential relevance on the toxicity severity. As dosimetry is not routinely conducted for these patients, this information was not integrated either. We also did not have extended follow-up beyond 6 months; thus, we were not able to include overall survival or progression-free survival in our analysis. This could have been interesting, especially as two factors associated with toxicity found in our study, namely, tumor grade and bone metastasis, were negatively associated with overall survival in a study on the efficacy of [^177^Lu]Lu-oxodotreotide.^26^

This monocentric and retrospective study focuses on a rare and very heterogeneous pathology, both in terms of physiopathology and management. [^177^Lu]Lu-oxodotreotide is a clinically and biologically well-tolerated therapy, and its safety profile must continue to be characterized to support its use as a first-line treatment, or as a retreatment.^27,28^ We believe that closer patient monitoring up to 6 months is required for biological parameter normalization and for possible semi-delayed toxicity detection. Beyond that, there seems to be an overabundance of confounding factors, such as a resumption of chemotherapy or disease relapse. The detection of delayed toxicity occurrence requires long-term follow-up as recommended in the SmPC, such as myelodysplastic syndrome, leukemia, or delayed nephropathy.^4,6,29,30^

## Conclusion

This study showed that the presence of bone metastases and less frequent metastases (peritoneal, pancreatic, or pulmonary), a higher tumor grade, and a gastrointestinal primary tumor were independent factors associated with hematological and hepatic toxicity during [^177^Lu]Lu-oxodotreotide treatment. Renal toxicity was very modest, and no associated factors could be identified. Permanent discontinuation of treatment after reversible toxicity results in a loss of chance for the patient, especially in the case of last-line or rescue treatment. Predicting toxicities by identifying their associated factors could encourage a wider use of [^177^Lu]Lu-oxodotreotide, with the aim of improving patient management by anticipating and preventing them. Further research is essential to validate these findings and explore the implications of identified risk factors on delayed toxicity and survival outcomes.

## Data Availability

The datasets generated during and/or analyzed during the current study are not publicly available due to their confidential nature but are available from the corresponding author on reasonable request.
