# Supplementary material for: Short-Term Biological Toxicity Prediction of [177Lu]Lutetium-Oxodotreotide: An Original Retrospective Analysis
Source: Cancer Biother Radiopharm. 2024 Jun 26;39(5):381–9. doi: 10.1089/cbr.2023.0195 (PMC11304756; doi:10.1089/cbr.2023.0195)
Supplement: Supplementary Figure S1 [file cbr.2023.0195_supplementary_information.pdf]

Short-term biological toxicity prediction of [^{177}Lu]Lutetium-oxodotreotide: an original retrospective analysis

Julien Dubois[1,2], Guillaume Tosato[3], Philippe Garrigue[1], David Taieb[4], Benjamin Guillet[1], Vincent Nail[1]

Supplementary Information

Biological toxicity grades were defined according to National Cancer Institute Common Terminology Criteria for Adverse Events (NCI CTCAE) version 5.0. Toxicities were staged based on their severity, from the lowest grade 1 (G1) to the highest grade 4 (G4). Studied parameters grades for the study are described in Supplementary table 1.

Supplementary table 1: Studied parameter grades following the CTCAE v5.0

Parameters (unit) *CTCAE Term*	*Grade 1: mild*	*Grade 2: moderate*	*Grade 3: severe*	*Grade 4: life-threatening*
Thrombocytes (/mm^3) *Platelet count decreased*	75,000 – LLN (100,000)	50,000 – 75,000	25,000 – 50,000	<25,000
Hemoglobin level (g/dL) *Anemia*	10.0 – LLN (11.5)	8.0 – 10.0	<8.0	Life-threatening consequences
Neutrophils (/mm^3) *Neutrophil count decreased*	1500 – LLN (2000)	1000 – 1500	500 – 1000	<500
Lymphocytes (/mm^3) *Lymphocyte count decreased*	800 – LLN (1000)	500 – 800	200 – 500	<200
Monocytes (/mm^3) *Not described yet*	350 – LLN (450)	250 – 350	150 – 250	<150
Creatininemia (µmol/L) *Creatinine increased*	ULN (110) – 1.5 x ULN	1.5 – 3 x ULN	3 - 6 x ULN	>6 x ULN
GFR decreased (mL/min/1.73m^2) *Chronic kidney disease*	60 – LLN (90)	30 - 60	15 - 30	<15
ALT (UI/L) *Alanine aminotransferase increased*	ULN (40) – 3 x ULN	3 – 5 x ULN	5 – 20 x ULN	>20 x ULN
AST (UI/L) *Aspartate aminotransferase increased*	ULN (35) – 3 x ULN	3 – 5 x ULN	5 – 20 x ULN	>20 x ULN
ALP (UI/L) *Alkaline phosphatase increased*	ULN (150) – 2.5 x ULN	2.5 – 5 x ULN	5 – 20 x ULN	>20 x ULN
GGT (UI/L) *Gamma-glutamyl transferase increased*	ULN (45) – 2.5 x ULN	2.5 – 5 x ULN	5 – 20 x ULN	>20 x ULN

CTCAE: Common terminology criteria for adverse events; LLN: Lower limit of normal; ULN: Upper limit of normal; GFR: Glomerular filtration rate; ALT: Alanine aminotransferase; AST: Aspartate aminotransferase; ALP: Alkaline phosphatase; GGT: Gamma-glutamyl transferase

Concerning PRRT, median period between initial diagnosis to treatment initiation was 6 years (IQR: 3 to 11 years). Chemotherapy and targeted therapy were stopped at least 1 month before PRRT. Long-acting somatostatin analogs were stopped one month (up to two weeks) before PRRT, while short-acting analogs were stopped 24 hours (up to 12 hours) before PRRT as specified in the recommendations. Thirty-seven patients (92.5%) received recommended therapeutic scheme of [^{177}Lu]Lu-oxodotreotide (median cumulative activity: 29.24 GBq ; IQR: 29.00 to 29.40 GBq). Dose reductions due to toxicity were required for 3 patients (7.5%) from the first, second and third cycles. After decreasing, doses were maintained to 3 700 MBq. Spacing up to 8 weeks between cycles was required for 8 patients (20.0%) due to toxicity occurrence, but not necessarily maintained thereafter (Supplementary table 2). These adjustments reflect the tailored approach taken to manage the treatment regimen in response to the adverse effects observed, ensuring patient safety and treatment efficacy.

Supplementary table 2: Characteristics of the administered doses (n=40)

Parameters	*Value*
**Infused activity (GBq),** median [IQR]	
Theoretical infused activity of 7.4 GBq (n=154)	7.32 [7.20 – 7.40]
Theoretical infused activity of 3.7 GBq (n=6)	3.63 [3.58 – 3.65]
**Regimen without dose reduction**, n (%)	37 (92.5%)
Cumulative infused activity (GBq), median [IQR]	29.24 [29.00 – 29.40]
**Regimen with dose reduction**, n (%)	3 (7.5%)
Cumulative infused activity (GBq)	
1 infusion to 3.7 GBq	25.7
2 infusions to 3.7 GBq	22.2
3 infusions to 3.7 GBq	17.9
**Cycle spacing**, n (%)	
Without dose reduction	6 (15,0%)
With dose reduction	2 (5,0%)

IQR: Interquartile range; GBq: GigaBecquerel

Supplementary table 3: Proportions of hematologic toxicity, (a) prior to PRRT, and (b) during PRRT (n=40)

a

	**Baseline disorders severity**			
	Grade 1	***Grade 2***	***Grade 3***	***Grade 4***
Thrombocytopenia	1 (2.5%)			
Anemia	3 (7.5%)			
Neutropenia	3 (7.5%)			
Lymphopenia		4 (10.0%)		
Monocytopenia	4 (10.0%)	1 (2.5%)		

b

	Toxicity during PRRT				
	Thrombocytopenia	***Anemia***	***Neutropenia***	***Lymphopenia***	***Monocytopenia***
Severity overall cycles					
Grade 1	2 (5.0%)	7 (17.5%)	7 (17.5%)	5 (12.5%)	9 (22.5%)
Grade 2	7 (17.5%)	9 (22.5%)	6 (15.0%)	12 (30.0%)	6 (15.0%)
Grade 3	3 (7.5%)	2 (5.0%)	5 (12.5%)	17 (42.5%)	7 (17.5%)
Grade 4	1 (2.5%)		1 (2.5%)	2 (5.0%)	2 (5.0%)
Total	13 (33.0%)	18 (45.0%)	19 (47.5%)	36 (90.0%)	24 (60.0%)
Toxicity incidence					
Cycle 1	5 (12.5%)	7 (17.5%)	9 (22.5%)	25 (62.5%)	16 (40.0%)
Cycle 2	1 (2.5%)	3 (7.5%)	4 (10.0%)	7 (17.5%)	5 (12.5%)
Cycle 3	3 (7.5%)	2 (5.0%)	4 (10.0%)	2 (5.0%)	
Cycle 4	3 (7.5%)	4 (10.0%)	1 (2.5%)	2 (5.0%)	2 (5.0%)
M3	1 (2.5%)				1 (2.5%)
M6		2 (5.0%)	1 (2.5%)		
Total	13 (33.0%)	18 (45.0%)	19 (47.5%)	36 (90.0%)	24 (60.0%)

PRRT: Peptide receptor radionuclide therapy

Supplementary table 4: Proportions of renal and liver toxicity grades, (a) prior to PRRT, and (b) during PRRT (n=40)

a

	Baseline disorders severity			
	Grade 1	***Grade 2***	***Grade 3***	***Grade 4***
GFR decreased	18 (45.0%)	4 (10.0%)		
Creatinine increased	4 (10.0%)			
AST increased	4 (10.0%)	3 (7.5%)	1 (2.5%)	
ALT increased	9 (22.5%)	3 (7.5%)	1 (2.5%)	
ALP increased	11 (27.5%)	2 (5.0%)	1 (2.5%)	
GGT increased	12 (30.0%)	3 (7.5%)	8 (20.0%)	

b

	Toxicity during PRRT					
	GFR decreased	***Creatinine increased***	***AST increased***	***ALT increased***	***ALP increased***	***GGT increased***
Severity overall cycles						
Grade 1	24 (60.0%)	8 (20.0%)	18 (45.0%)	15 (37.5%)	11 (27.5%)	14 (35.0%)
Grade 2	12 (30.0%)	2 (5.0%)	3 (7.5%)	4 (10.0%)	5 (12.5%)	3 (7.5%)
Grade 3			2 (5.0%)	3 (7.5%)	3 (7.5%)	12 (30.0%)
Grade 4	1 (2.5%)					3 (7.5%)
Total	37 (92.5%)	10 (25.0%)				
Toxicity incidence						
Cycle 1	30 (75.0%)	5 (12.5%)	17 (42.5%)	16 (40.0%)	16 (40.0%)	27 (67.5%)
Cycle 2	4 (10.0%)	3 (7.5%)	3 (7.5%)	2 (5.0%)	1 (2.5%)	1 (2.5%)
Cycle 3	2 (5.0%)		3 (7.5%)	1 (2.5%)	1 (2.5%)	1 (2.5%)
Cycle 4		1 (2.5%)		1 (2.5%)		2 (5.0%)
M3	1 (2.5%)			1 (2.5%)	11 (2.5%)	1 (2.5%)
M6		1 (2.5%)		1 (2.5%)		
Total	37 (92.5%)	10 (25.0%)				

PRRT: Peptide receptor radionuclide therapy; GFR: Glomerular filtration rate; ALT: Alanine aminotransferase; AST: Aspartate aminotransferase; ALP: Alkaline phosphatase; GGT: Gamma-glutamyl transferase

Supplementary figure 1: Factors associated with grade 2 thrombocytopenia (< 75,000/mm^3)

Parameters	OR	95%CI	P
Male_gender	1.0	[0.32-3.4]	0.94
Age	1.0	[0.96-1.1]	0.64
Weight	0.98	[0.95-1.0]	0.41
BMI	1.0	[0.86-1.2]	0.86
Cardiovascular_comorbidities	0.54	[0.16-1.8]	0.32
Other_comorbidities	1.5	[0.40-5.7]	0.54
Pancreatic_NEN	0.78	[0.19-3.3]	0.73
Other_NEN	1.2	[0.27-4.9]	0.84
Tumor_grade	1.7	[0.45-6.5]	0.43
Liver_metastasis	0.60	[0.17-2.1]	0.43
Lymph_node_metastasis	0.66	[0.19-2.3]	0.51
Bone_metastasis	3.3	[1.0-11]	**0.047**
Peritoneal_metastasis	2.2	[0.57-8.2]	0.25
Other_metastasis	2.5	[0.72-8.6]	0.15
Previous_somatostatin_analog	1.3	[0.34-4.9]	0.70
Previous_immunosuppressive_agent	0.57	[0.15-2.1]	0.40
Previous_sunitinib	0.34	[0.040-2.7]	0.30
Previous_cytotoxic_chemotherapy	0.92	[0.27-3.2]	0.89
Previous_hepatic_chemoembolization	2.2	[0.68-7.3]	0.19
Previous_surgical_resection	1.1	[0.28-4.1]	0.92
Previous_external_radiotherapy	1.6	[0.43-6.1]	0.48
LysArg_infusion	0.91	[0.24-3.5]	0.89
G1_Thrombocytopenia_before	1.4	[3.7-55]	**< 0.001**
G1_Anemia_before	1.5	[0.47-5.1]	0.48
G1_Neutropenia_before	4.7	[1.0-22]	**0.049**
G1_Lymphopenia_before	1.1	[0.14-8.6]	0.93
G1_Monocytopenia_before	3.2	[0.69-15]	0.14
G2_Altered_GFR_before	0.71	[0.19-2.7]	0.61
G1_ALT_increased_before	0.44	[0.060-3.5]	0.44

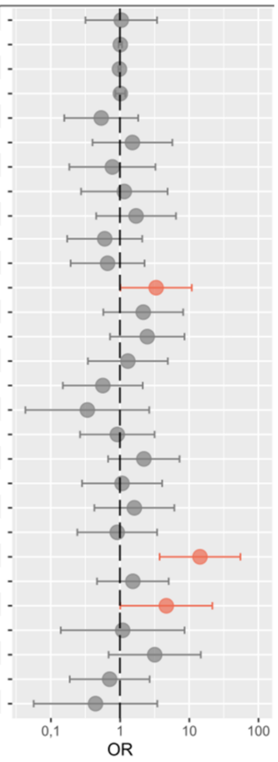

OR: Odds ratio; 95%CI: 95% Confidence interval; P: Wald test P-value; G: Grade; BMI: Body mass index; NEN: Neuroendocrine neoplasm; LysArg: Lysine-arginine solution; GFR: Glomerular filtration rate; ALT: Alanine aminotransferase

Supplementary figure 2: Factors associated with grade 2 anemia (< 10.0 g/dL)

Parameters	OR	95%CI	P
Male_gender	0.53	[0.16-1.8]	0.30
Age	1.0	[0.98-1.1]	0.26
Weight	0.93	[0.88-0.98]	**0.003**
BMI	0.72	[0.57-0.92]	**0.008**
Cardiovascular_comorbidities	0.33	[0.090-1.2]	0.10
Other_comorbidities	1.2	[0.34-4.0]	0.82
Pancreatic_NEN	0.13	[0.020-1.1]	0.057
Other_NEN	0.45	[0.090-2.1]	0.31
Tumor_grade	3.0	[0.64-14]	0.16
Liver_metastasis	1.7	[0.37-8.0]	0.49
Lymph_node_metastasis	0.23	[0.050-1.1]	0.063
Bone_metastasis	2.3	[0.70-7.7]	0.17
Peritoneal_metastasis	4.4	[1.2-16]	**0.023**
Other_metastasis	1.2	[0.31-4.6]	0.79
Previous_somatostatin_analog	1.1	[0.30-4.3]	0.85
Previous_immunosuppressive_agent	1.3	[0.39-4.2]	0.68
Previous_sunitinib	0.35	[0.040-2.7]	0.32
Previous_cytotoxic_chemotherapy	1.3	[0.40-4.4]	0.65
Previous_hepatic_chemoembolization	2.1	[0.64-7.0]	0.22
Previous_surgical_resection	1.2	[0.30-5.1]	0.78
Previous_external_radiotherapy	1.0	[0.22-4.8]	0.96
LysArg_infusion	0.86	[0.23-3.3]	0.83
G1_Thrombocytopenia_before	1.2	[0.33-4.7]	0.75
G1_Anemia_before	9.3	[2.0-43]	**0.004**
G1_Neutropenia_before	2.9	[0.77-11]	0.12
G1_Lymphopenia_before	0.55	[0.12-2.6]	0.45
G1_Monocytopenia_before	1.6	[0.42-6.0]	0.50
G2_Altered_GFR_before	2.2	[0.68-7.4]	0.19
G1_ALT_increased_before	1.2	[0.26-5.8]	0.79

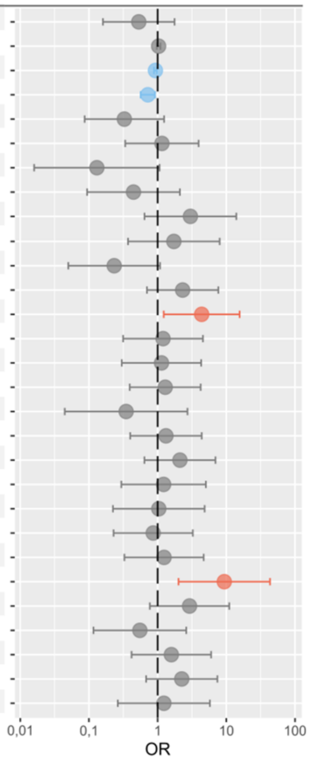

OR: Odds ratio; 95%CI: 95% Confidence interval; P: Wald test P-value; G: Grade; BMI: Body mass index; NEN: Neuroendocrine neoplasm; LysArg: Lysine-arginine solution; GFR: Glomerular filtration rate; ALT: Alanine aminotransferase

No risk factor associated with the occurrence of G1 neutropenia or G1 lymphopenia was identified (Supplementary figure 3a and 4a). Previous G1 thrombocytopenia was found as a G2 neutropenia associated risk factor (OR 3.2, p=0.045, Supplementary figure 3b). High weight and high BMI were found as G2 neutropenia protective factors (respectively OR 0.95, p=0.020, and OR 0.66, p=0.006, Supplementary figure 3b). Three variables were significant protective factors of G2 lymphopenia: pancreatic origin NEN, previous treatment with sunitinib, and having a G1 lymphopenia (respectively OR 0.26, p=0.009, OR 0.26, p=0.027, and OR 0.36, p=0.007, Supplementary figure 4b). Pancreatic origin NEN was still found as an associated G3 lymphopenia protective factor (OR 0.15, p=0.013, Supplementary figure 4c). Previous hepatic chemoembolization was identified as an associated G3 lymphopenia risk factor (OR 3.2, p=0.013, Supplementary figure 4c). Finally, bone metastases were identified as G1 and G3 monocytopenia associated risk factor (respectively OR 2.6, p=0.020, and OR 0.11, p=0.039, Supplementary figure 5a and 5c). Lymph node metastases was found as G2 and G3 monocytopenia associated protective factor (respectively OR 0.20, p=0.067, and OR 0.11, p<0.034, Supplementary figure 5b and 5c).

Supplementary figure 3: Factors associated with (a) grade 1 neutropenia (< 2000/mm^3), and (b) grade 2 neutropenia (< 1500/mm^3)

a

Parameters	OR	95%CI	P
Male_gender	0.42	[0.18-1.0]	0.053
Age	1.0	[0.97-1.1]	0.52
Weight	0.98	[0.96-1.0]	0.17
BMI	1.0	[0.90-1.1]	0.95
Cardiovascular_comorbidities	0.32	[0.12-0.83]	0.019
Other_comorbidities	0.73	[0.31-1.7]	0.48
Pancreatic_NEN	0.83	[0.31-2.2]	0.71
Other_NEN	0.67	[0.21-2.1]	0.49
Tumor_grade	1.4	[0.55-3.4]	0.51
Liver_metastasis	2.2	[0.73-6.5]	0.17
Lymph_node_metastasis	0.50	[0.20-1.2]	0.13
Bone_metastasis	2.1	[0.85-5.0]	0.11
Peritoneal_metastasis	2.1	[0.78-5.9]	0.14
Other_metastasis	1.6	[0.60-4.0]	0.37
Previous_somatostatin_analog	0.95	[0.37-2.4]	0.91
Previous_immunosuppressive_agent	0.71	[0.28-1.8]	0.46
Previous_sunitinib	1.1	[0.38-3.3]	0.84
Previous_cytotoxic_chemotherapy	1.4	[0.59-3.4]	0.44
Previous_hepatic_chemoembolization	1.5	[0.63-3.7]	0.35
Previous_surgical_resection	0.63	[0.25-1.6]	0.32
Previous_external_radiotherapy	0.93	[0.31-2.8]	0.90
LysArg_infusion	0.83	[0.32-2.1]	0.70
G1_Thrombocytopenia_before	1.0	[0.30-3.4]	0.99
G1_Anemia_before	0.93	[0.31-2.8]	0.90
G1_Lymphopenia_before	0.08	[0.030-0.22]	< 0.001
G1_Monocytopenia_before	1.5	[0.62-3.5]	0.39
G2_Altered_GFR_before	0.95	[0.35-2.6]	0.92
G1_ALT_increased_before	0.53	[0.12-2.3]	0.39

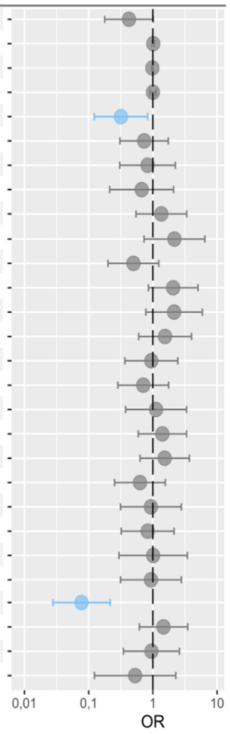

b

Parameters	OR	95%CI	P
Male_gender	0.74	[0.24-2.3]	0.60
Age	1.0	[0.98-1.1]	0.24
Weight	0.95	[0.92-0.99]	**0.020**
BMI	0.66	[0.49-0.89]	**0.006**
Cardiovascular_comorbidities	0.29	[0.08-1.1]	0.064
Other_comorbidities	0.67	[0.21-2.1]	0.49
Pancreatic_NEN	0.98	[0.27-3.5]	0.97
Other_NEN	0.59	[0.12-2.9]	0.51
Tumor_grade	2.0	[0.53-7.3]	0.31
Liver_metastasis	2.1	[0.45-9.5]	0.35
Lymph_node_metastasis	0.37	[0.10-1.4]	0.13
Bone_metastasis	1.9	[0.60-6.0]	0.28
Peritoneal_metastasis	1.9	[0.51-7.1]	0.33
Other_metastasis	3.0	[0.95-9.4]	0.062
Previous_somatostatin_analog	0.93	[0.28-3.1]	0.91
Previous_immunosuppressive_agent	0.51	[0.14-1.9]	0.32
Previous_sunitinib	0.83	[0.18-3.8]	0.81
Previous_cytotoxic_chemotherapy	1.8	[0.56-5.5]	0.33
Previous_hepatic_chemoembolization	1.7	[0.54-5.3]	0.37
Previous_surgical_resection	1.2	[0.31-4.3]	0.82
Previous_external_radiotherapy	0.73	[0.16-3.3]	0.68
LysArg_infusion	1.8	[0.40-8.3]	0.44
G1_Thrombocytopenia_before	3.2	[1.0-9.91]	**0.045**
G1_Anemia_before	2.3	[0.74-7.2]	0.15
G1_Neutropenia_before	2.2	[0.70-7.0]	0.18
G1_Lymphopenia_before	0.29	[0.080-1.1]	0.064
G1_Monocytopenia_before	2.3	[0.61-8.4]	0.22
G2_Altered_GFR_before	0.48	[0.10-2.2]	0.34
G1_ALT_increased_before	0.39	[0.050-3.1]	0.37

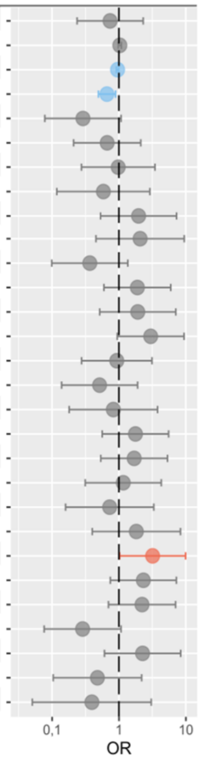

OR: Odds ratio; 95%CI: 95% Confidence interval; P: Wald test P-value; G: Grade; BMI: Body mass index; NEN: Neuroendocrine neoplasm; LysArg: Lysine-arginine solution; GFR: Glomerular filtration rate; ALT: Alanine aminotransferase

Supplementary figure 4: Factors associated with (a) grade 1 lymphopenia (< 1000/mm^3), (b) grade 2 lymphopenia (< 800/mm^3), and (c) grade 3 lymphopenia (< 500/mm^3)

a

Parameters	OR	95%CI	P
Male_gender	1.8	[0.90-3.6]	0.095
Age	1.0	[0.98-1.0]	0.74
Weight	0.99	[0.98-1.0]	0.45
BMI	0.95	[0.88-1.0]	0.13
Cardiovascular_comorbidities	1.6	[0.83-3.1]	0.15
Other_comorbidities	1.6	[0.80-3.4]	0.18
Pancreatic_NEN	0.45	[0.19-1.0]	0.059
Other_NEN	1.2	[0.54-2.8]	0.63
Tumor_grade	1.0	[0.51-2.0]	0.98
Liver_metastasis	0.85	[0.41-1.8]	0.66
Lymph_node_metastasis	0.86	[0.44-1.7]	0.66
Bone_metastasis	0.68	[0.33-1.4]	0.31
Peritoneal_metastasis	0.70	[0.27-1.8]	0.46
Other_metastasis	1.1	[0.50-2.4]	0.81
Previous_somatostatin_analog	1.3	[0.62-2.7]	0.51
Previous_immunosuppressive_agent	1.5	[0.76-2.9]	0.25
Previous_sunitinib	0.51	[0.21-1.3]	0.14
Previous_cytotoxic_chemotherapy	0.93	[0.47-1.9]	0.84
Previous_hepatic_chemoembolization	1.8	[0.88-3.7]	0.11
Previous_surgical_resection	1.1	[0.53-2.4]	0.75
Previous_external_radiotherapy	1.5	[0.65-3.5]	0.34
LysArg_infusion	1.0	[0.48-2.1]	0.98
G1_Thrombocytopenia_before	0	[0-Inf]	1.0
G1_Anemia_before	0.49	[0.15-1.6]	0.24
G1_Neutropenia_before	0.27	[0.12-0.63]	**0.002**
G1_Monocytopenia_before	0.69	[0.35-1.4]	0.28
G2_Altered_GFR_before	0.73	[0.30-1.8]	0.48
G1_ALT_increased_before	0.47	[0.14-1.6]	0.22

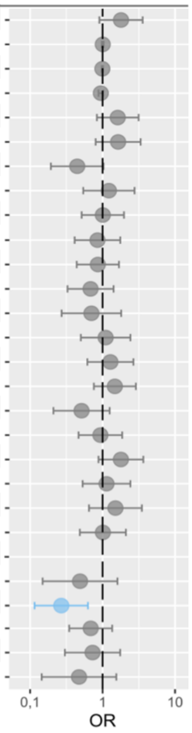

b

Parameters	OR	95%CI	P
Male_gender	1.6	[0.75-3.2]	0.24
Age	0.99	[0.96-1.0]	0.65
Weight	1.0	[0.98-1.0]	0.70
BMI	0.94	[0.86-1.0]	0.21
Cardiovascular_comorbidities	1.2	[0.61-2.5]	0.55
Other_comorbidities	1.9	[0.87-4.2]	0.11
Pancreatic_NEN	0.26	[0.090-0.71]	**0.009**
Other_NEN	1.1	[0.47-2.4]	0.88
Tumor_grade	1.4	[0.67-3.1]	0.35
Liver_metastasis	1.0	[0.47-2.2]	0.98
Lymph_node_metastasis	0.67	[0.32-1.4]	0.29
Bone_metastasis	0.66	[0.29-1.5]	0.31
Peritoneal_metastasis	1.1	[0.40-2.8]	0.92
Other_metastasis	1.3	[0.59-3.0]	0.51
Previous_somatostatin_analog	1.2	[0.55-2.6]	0.64
Previous_immunosuppressive_agent	1.2	[0.56-2.4]	0.69
Previous_sunitinib	0.26	[0.080-0.85]	**0.027**
Previous_cytotoxic_chemotherapy	0.67	[0.31-1.5]	0.33
Previous_hepatic_chemoembolization	1.7	[0.81-3.6]	0.16
Previous_surgical_resection	1.4	[0.58-3.2]	0.49
Previous_external_radiotherapy	1.5	[0.67-3.5]	0.31
LysArg_infusion	1.3	[0.59-3.0]	0.49
G1_Thrombocytopenia_before	0.31	[0.070-1.3]	0.11
G1_Anemia_before	0.52	[0.18-1.5]	0.23
G1_Neutropenia_before	0.48	[0.21-1.1]	0.074
G1_Lymphopenia_before	0.36	[0.17-0.76]	**0.007**
G1_Monocytopenia_before	0.52	[0.25-1.1]	0.083
G2_Altered_GFR_before	0.92	[0.41-2.1]	0.84
G1_ALT_increased_before	0.76	[0.26-2.2]	0.61

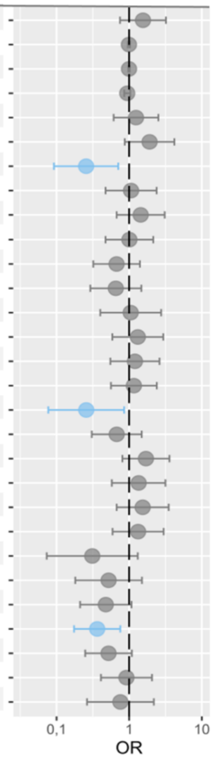

c

Parameters	OR	95%CI	P
Male_gender	1.4	[0.54-3.5]	0.50
Age	0.99	[0.95-1.0]	0.62
Weight	1.0	[0.97-1.0]	0.76
BMI	0.95	[0.84-1.1]	0.47
Cardiovascular_comorbidities	1.4	[0.56-3.5]	0.47
Other_comorbidities	1.4	[0.53-3.7]	0.51
Pancreatic_NEN	0.15	[0.030-0.67]	**0.013**
Other_NEN	0.60	[0.21-1.7]	0.33
Tumor_grade	1.2	[0.48-3.1]	0.68
Liver_metastasis	1.3	[0.47-3.6]	0.62
Lymph_node_metastasis	0.64	[0.25-1.6]	0.35
Bone_metastasis	0.82	[0.29-2.3]	0.70
Peritoneal_metastasis	1.9	[0.63-5.8]	0.25
Other_metastasis	2.0	[0.75-5.3]	0.16
Previous_somatostatin_analog	1.3	[0.47-3.6]	0.60
Previous_immunosuppressive_agent	1.5	[0.60-3.7]	0.39
Previous_sunitinib	0.14	[0.020-1.1]	0.060
Previous_cytotoxic_chemotherapy	0.98	[0.39-2.5]	0.97
Previous_hepatic_chemoembolization	3.2	[1.3-7.9]	**0.013**
Previous_surgical_resection	1.5	[0.49-4.4]	0.50
Previous_external_radiotherapy	1.0	[0.34-3.1]	0.98
LysArg_infusion	1.0	[0.37-2.9]	0.96
G1_Thrombocytopenia_before	0.85	[0.25-2.9]	0.80
G1_Anemia_before	0.78	[0.28-2.2]	0.63
G1_Neutropenia_before	0.41	[0.15-1.2]	0.091
G1_Lymphopenia_before	2.7	[0.37-21]	0.33
G1_Monocytopenia_before	0.65	[0.26-1.6]	0.34
G2_Altered_GFR_before	0.82	[0.29-2.3]	0.70
G1_ALT_increased_before	1.0	[0.30-3.6]	0.95

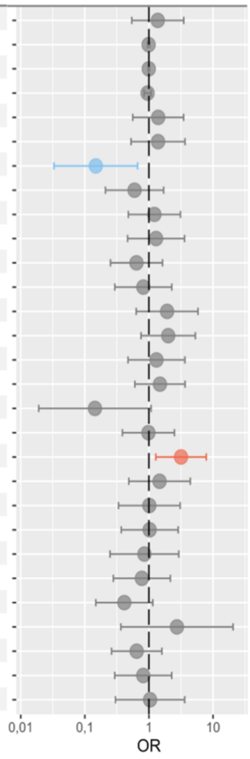

OR: Odds ratio; 95%CI: 95% Confidence interval; P: Wald test P-value; G: Grade; BMI: Body mass index; NEN: Neuroendocrine neoplasm; LysArg: Lysine-arginine solution; GFR: Glomerular filtration rate; ALT: Alanine aminotransferase

Supplementary figure 5: Factors associated with (a) grade 1 monocytopenia (< 450/mm^3), (b) grade 2 monocytopenia (< 350/mm^3), and (c) grade 3 monocytopenia (< 250/mm^3)

a

Parameters	OR	95%CI	P
Male_gender	0.64	[0.29-1.4]	0.26
Age	1.0	[0.97-1.0]	0.90
Weight	0.99	[0.97-1.0]	0.56
BMI	0.98	[0.88-1.1]	0.75
Cardiovascular_comorbidities	0.60	[0.27-1.4]	0.22
Other_comorbidities	1.2	[0.52-2.7]	0.68
Pancreatic_NEN	0.86	[0.34-2.1]	0.74
Other_NEN	1.1	[0.39-2.9]	0.92
Tumor_grade	0.81	[0.36-1.8]	0.61
Liver_metastasis	1.9	[0.72-5.1]	0.20
Lymph_node_metastasis	0.63	[0.28-1.4]	0.27
Bone_metastasis	2.6	[1.2-5.7]	**0.020**
Peritoneal_metastasis	1.1	[0.36-3.1]	0.91
Other_metastasis	1.2	[0.46-2.9]	0.76
Previous_somatostatin_analog	0.85	[0.37-2.0]	0.71
Previous_immunosuppressive_agent	0.79	[0.35-1.8]	0.57
Previous_sunitinib	0.88	[0.33-2.4]	0.80
Previous_cytotoxic_chemotherapy	1.5	[0.66-3.2]	0.36
Previous_hepatic_chemoembolization	1.4	[0.63-3.3]	0.39
Previous_surgical_resection	0.96	[0.40-2.3]	0.93
Previous_external_radiotherapy	1.4	[0.53-3.8]	0.49
LysArg_infusion	1.2	[0.47-3.0]	0.72
G1_Thrombocytopenia_before	0.32	[0.040-2.4]	0.27
G1_Anemia_before	0.40	[0.12-1.4]	0.14
G1_Neutropenia_before	0.53	[0.16-1.8]	0.31
G1_Lymphopenia_before	0.14	[0.060-0.35]	**< 0.001**
G2_Altered_GFR_before	0.15	[0.020-1.1]	0.060
G1_ALT_increased_before	0.19	[0.030-1.4]	0.11

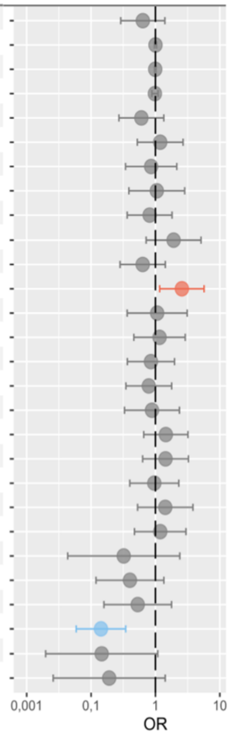

b

Parameters	OR	95%CI	P
Male_gender	0.71	[0.26-1.9]	0.49
Age	1.0	[0.97-1.1]	0.68
Weight	0.99	[0.96-1.0]	0.59
BMI	0.96	[0.83-1.1]	0.65
Cardiovascular_comorbidities	0.72	[0.27-1.9]	0.52
Other_comorbidities	1.9	[0.62-6.0]	0.26
Pancreatic_NEN	0.31	[0.06-1.5]	0.14
Other_NEN	1.6	[0.54-4.5]	0.41
Tumor_grade	0.94	[0.34-2.6]	0.91
Liver_metastasis	0.90	[0.31-2.6]	0.85
Lymph_node_metastasis	0.20	[0.060-0.72]	**0.014**
Bone_metastasis	2.5	[0.94-6.8]	0.067
Peritoneal_metastasis	1.5	[0.41-5.2]	0.56
Other_metastasis	1.2	[0.37-3.6]	0.80
Previous_somatostatin_analog	0.75	[0.27-2.1]	0.59
Previous_immunosuppressive_agent	0.68	[0.24-2.0]	0.47
Previous_sunitinib	0.90	[0.26-3.2]	0.87
Previous_cytotoxic_chemotherapy	1.0	[0.37-2.9]	0.95
Previous_hepatic_chemoembolization	1.2	[0.41-3.4]	0.76
Previous_surgical_resection	3.0	[0.68-13]	0.15
Previous_external_radiotherapy	2.6	[0.90-7.8]	0.079
LysArg_infusion	1.1	[0.36-3.4]	0.86
G1_Thrombocytopenia_before	0.76	[0.17-3.3]	0.71
G1_Anemia_before	0.37	[0.080-1.6]	0.19
G1_Neutropenia_before	0.52	[0.15-1.8]	0.31
G1_Lymphopenia_before	0.11	[0.040-0.31]	**< 0.001**
G1_Monocytopenia_before	0.74	[0.27-2.1]	0.56
G2_Altered_GFR_before	0.44	[0.10-2.0]	0.28
G1_ALT_increased_before	0	[0-Inf]	1.0

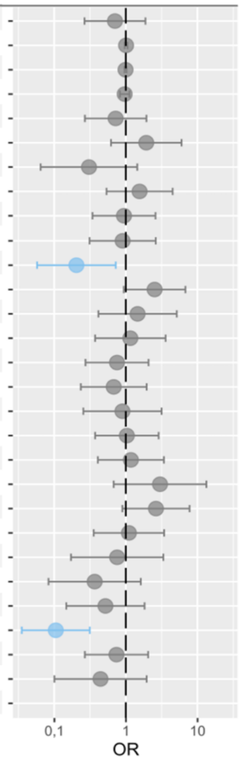

C

Parameters	OR	95%CI	P
Male_gender	0.85	[0.26-2.8]	0.79
Age	1.0	[0.97-1.1]	0.31
Weight	0.97	[0.94-1.0]	0.16
BMI	0.83	[0.63-1.1]	0.17
Cardiovascular_comorbidities	0.56	[0.16-1.9]	0.35
Other_comorbidities	1.5	[0.39-5.6]	0.56
Pancreatic_NEN	0.24	[0.030-2.0]	0.19
Other_NEN	1.4	[0.41-5.1]	0.57
Tumor_grade	1.1	[0.33-3.8]	0.86
Liver_metastasis	1.1	[0.28-4.1]	0.92
Lymph_node_metastasis	0.11	[0.010-0.84]	**0.034**
Bone_metastasis	3.5	[1.1-12]	**0.039**
Peritoneal_metastasis	2.1	[0.56-8.0]	0.27
Other_metastasis	1.5	[0.39-5.6]	0.57
Previous_somatostatin_analog	1.1	[0.28-4.0]	0.93
Previous_immunosuppressive_agent	0.87	[0.25-3.0]	0.82
Previous_sunitinib	1.0	[0.22-4.7]	1.0
Previous_cytotoxic_chemotherapy	1.5	[0.47-5.0]	0.48
Previous_hepatic_chemoembolization	2.0	[0.59-6.4]	0.27
Previous_surgical_resection	3.7	[0.47-29]	0.21
Previous_external_radiotherapy	2.6	[0.76-9.0]	0.13
LysArg_infusion	0.98	[0.26-3.7]	0.98
G1_Thrombocytopenia_before	1.5	[0.39-5.6]	0.56
G1_Anemia_before	0.75	[0.20-2.8]	0.67
G1_Neutropenia_before	0.57	[0.15-2.2]	0.41
G1_Lymphopenia_before	0.12	[0.040-0.41]	**0.001**
G1_Monocytopenia_before	1.4	[0.41-4.8]	0.59
G2_Altered_GFR_before	0.34	[0.040-2.7]	0.31
G1_ALT_increased_before	0	[0-Inf]	1.0

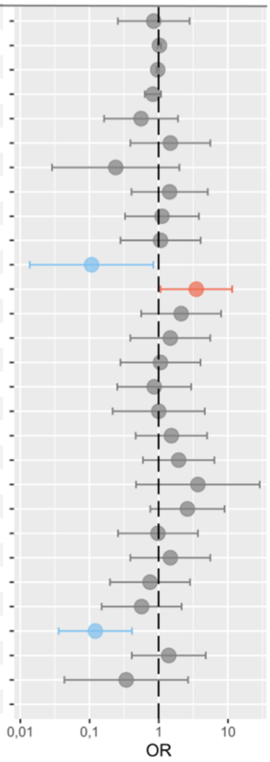

OR: Odds ratio; 95%CI: 95% Confidence interval; P: Wald test P-value; G: Grade; BMI: Body mass index; NEN: Neuroendocrine neoplasm; LysArg: Lysine-arginine solution; GFR: Glomerular filtration rate; ALT: Alanine aminotransferase

Presence of peritoneal metastases was identified as G1 AST increased, and G1 ALT increased associated risk factor (respectively OR 7.0, p=0.018, and OR 4.5, p=0.048, Supplementary figure 6a and 6b). Another revealed associated risk factor of G1 ALT increase was the presence of other metastases (OR 5.5, p=0.026). Previous surgical resection was established as an associated protective factor for G1 ALT increase (OR 0.21, p=0.038, Supplementary figure 6b). Hepatic chemoembolization was determined as an associated G1 ALP increase and G1 GGT increase risk factor (respectively OR 22, p=0.004, and OR 5.1, p<0.001, Supplementary figure 6c and 6d). Presence of liver metastases, bone metastases and other metastases, as well as a cytotoxic chemotherapy treatment prior to PRRT, were identified as associated risk factors for G1 GGT increase (respectively OR 4.5, p=0.047, OR 3.9, p=0.004, OR 2.6, p=0.048, and OR 2.8, p=0.033, Supplementary figure 6d) but not as associated risk factors G1 ALP increase.

Supplementary figure 6: Factors associated with (a) grade 2 AST increase (> 3xULN), (b) grade 2 ALT increase (> 3xULN), (c) grade 2 GGT increase (> 2.5xULN), and (d) grade 2 ALP increase (> 2.5xULN)

a

Parameters	OR	95%CI	P
Male_gender	3.9	[0.46-34]	0.21
Age	0.95	[0.89-1.0]	0.13
Weight	1.0	[0.96-1.1]	0.92
BMI	0.92	[0.76-1.1]	0.38
Cardiovascular_comorbidities	0.18	[0.020-1.6]	0.12
Other_comorbidities	1.2	[0.21-6.3]	0.87
Pancreatic_NEN	1.1	[0.18-6.3]	0.96
Other_NEN	0.56	[0.060-5.4]	0.62
Tumor_grade	Inf	[0-Inf]	1.0
Liver_metastasis	Inf	[0-Inf]	1.0
Lymph_node_metastasis	3.1	[0.57-17]	0.19
Bone_metastasis	2.3	[0.47-12]	0.31
Peritoneal_metastasis	7.0	[1.4-35]	**0.018**
Other_metastasis	2.0	[0.36-11]	0.44
Previous_somatostatin_analog	Inf	[0-Inf]	1.0
Previous_immunosuppressive_agent	0.71	[0.13-3.9]	0.69
Previous_sunitinib	0.74	[0.090-6.3]	0.78
Previous_cytotoxic_chemotherapy	0.82	[0.15-4.5]	0.82
Previous_hepatic_chemoembolization	1.1	[0.21-6.2]	0.89
Previous_surgical_resection	0.30	[0.060-1.5]	0.14
Previous_external_radiotherapy	0.74	[0.090-6.3]	0.78
LysArg_infusion	2.1	[0.24-18]	0.50
G1_Thrombocytopenia_before	0	[0-Inf]	1.0
G1_Anemia_before	0	[0-Inf]	1.0
G1_Neutropenia_before	0.22	[0.030-1.8]	0.16
G1_Lymphopenia_before	0.070	[0.010-0.41]	**0.003**
G1_Monocytopenia_before	0.34	[0.060-1.9]	0.22
G2_Altered_GFR_before	0.38	[0.040-3.2]	0.37
G1_ALT_increased_before	2.3	[0.27-20]	0.45

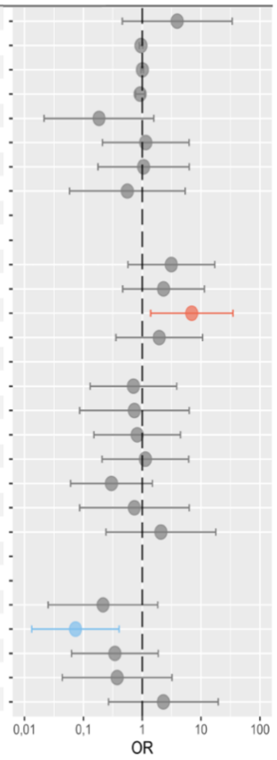

b

Parameters	OR	95%CI	P
Male_gender	2,0	[0,39-10]	0,41
Age	0,94	[0,89-1,0]	0,050
Weight	1,0	[0,97-1,1]	0,66
BMI	0,99	[0,86-1,1]	0,91
Cardiovascular_comorbidities	0,37	[0,070-1,9]	0,24
Other_comorbidities	0,77	[0,17-3,5]	0,74
Pancreatic_NEN	1,1	[0,18-6,5]	0,92
Other_NEN	1,3	[0,21-7,6]	0,80
Tumor_grade	Inf	[0-Inf]	1.0
Liver_metastasis	2,8	[0,34-23]	0,34
Lymph_node_metastasis	1,1	[0,25-4,9]	0,90
Bone_metastasis	1,9	[0,41-8,3]	0,42
Peritoneal_metastasis	4,5	[1,0-20]	**0,048**
Other_metastasis	5,5	[1,2-25]	**0,026**
Previous_somatostatin_analog	2,8	[0,34-23]	0,34
Previous_immunosuppressive_agent	1,1	[0,25-5,0]	0,88
Previous_sunitinib	0,66	[0,080-5,5]	0,70
Previous_cytotoxic_chemotherapy	0,69	[0,13-3,6]	0,66
Previous_hepatic_chemoembolization	1,8	[0,40-8,1]	0,44
Previous_surgical_resection	0,21	[0,050-0,92]	**0,038**
Previous_external_radiotherapy	1,7	[0,33-8,7]	0,54
LysArg_infusion	0,98	[0,19-5,1]	0,98
G1_Thrombocytopenia_before	0	[0-Inf]	1.0
G1_Anemia_before	0	[0-Inf]	1.0
G1_Neutropenia_before	0,19	[0,020-1,6]	0,12
G1_Lymphopenia_before	0,070	[0,010-0,36]	**0,002**
G1_Monocytopenia_before	0,25	[0,050-1,3]	0,10
G2_Altered_GFR_before	0,31	[0,040-2,5]	0,27

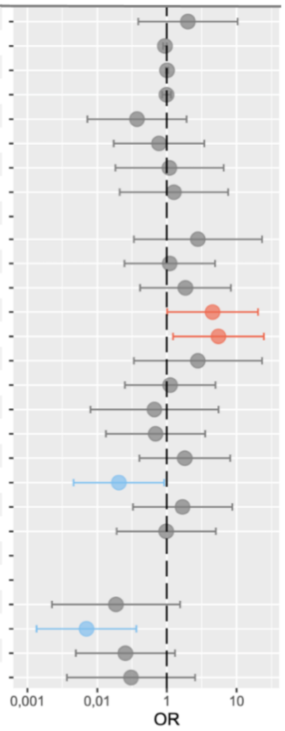

c

Parameters	OR	95%CI	P
Male_gender	1.1	[0.25-4.9]	0.90
Age	0.99	[0.93-1.1]	0.74
Weight	0.97	[0.93-1.0]	0.21
BMI	0.93	[0.75-1.1]	0.47
Cardiovascular_comorbidities	0.31	[0.060-1.6]	0.15
Other_comorbidities	1.0	[0.24-4.3]	0.98
Pancreatic_NEN	0.46	[0.090-2.4]	0.36
Other_NEN	0.32	[0.040-2.8]	0.30
Tumor_grade	1.8	[0.37-9.1]	0.46
Liver_metastasis	Inf	[0-Inf]	1.0
Lymph_node_metastasis	1.1	[0.26-4.5]	0.91
Bone_metastasis	2.2	[0.55-9.1]	0.26
Peritoneal_metastasis	2.3	[0.44-12]	0.33
Other_metastasis	1.9	[0.43-8.1]	0.41
Previous_somatostatin_analog	Inf	[0-Inf]	1.0
Previous_immunosuppressive_agent	3.0	[0.71-13]	0.14
Previous_sunitinib	0.65	[0.080-5.4]	0.69
Previous_cytotoxic_chemotherapy	1.7	[0.42-6.7]	0.47
Previous_hepatic_chemoembolization	22	[2.7-177]	**0.004**
Previous_surgical_resection	0.23	[0.050-1.0]	0.054
Previous_external_radiotherapy	0.56	[0.070-4.6]	0.59
LysArg_infusion	0.57	[0.14-2.4]	0.44
G1_Thrombocytopenia_before	0.44	[0.050-3.5]	0.44
G1_Anemia_before	0.71	[0.14-3.5]	0.68
G1_Neutropenia_before	1.1	[0.27-4.4]	0.90
G1_Lymphopenia_before	0.030	[0.0-0.25]	**0.001**
G1_Monocytopenia_before	0.59	[0.15-2.4]	0.46
G2_Altered_GFR_before	0.74	[0.15-3.7]	0.71
G1_ALT_increased_before	1.1	[0.13-8.8]	0.96

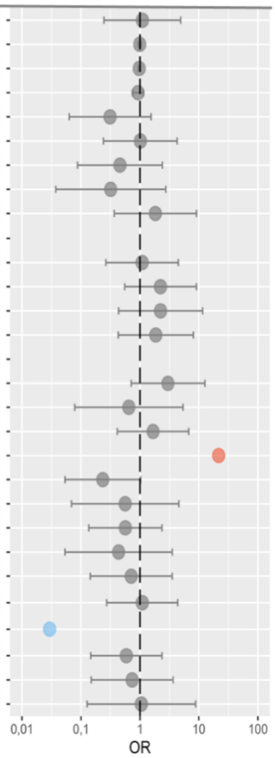

d

Parameters	OR	95%CI	P
Male_gender	1.8	[0.66-4.7]	0.26
Age	0.99	[0.95-1.0]	0.72
Weight	0.99	[0.96-1.0]	0.38
BMI	0.96	[0.86-1.1]	0.46
Cardiovascular_comorbidities	0.42	[0.16-1.1]	0.082
Other_comorbidities	1.1	[0.41-2.7]	0.93
Pancreatic_NEN	1.4	[0.51-3.9]	0.50
Other_NEN	0.65	[0.17-2.4]	0.52
Tumor_grade	1.3	[0.47-3.4]	0.64
Liver_metastasis	4.5	[1.0-19]	**0.047**
Lymph_node_metastasis	1.1	[0.43-2.7]	0.87
Bone_metastasis	3.9	[1.5-9.9]	**0.004**
Peritoneal_metastasis	1.6	[0.54-5.0]	0.38
Other_metastasis	2.6	[1.0-6.8]	**0.048**
Previous_somatostatin_analog	2.7	[0.78-9.3]	0.12
Previous_immunosuppressive_agent	1.9	[0.73-4.7]	0.19
Previous_sunitinib	1.3	[0.42-3.9]	0.65
Previous_cytotoxic_chemotherapy	2.8	[1.1-7.1]	**0.033**
Previous_hepatic_chemoembolization	5.1	[2.0-13]	**< 0.001**
Previous_surgical_resection	0.46	[0.18-1.2]	0.11
Previous_external_radiotherapy	0.81	[0.23-2.8]	0.74
LysArg_infusion	0.91	[0.32-2.6]	0.86
G1_Thrombocytopenia_before	0	[0-Inf]	1.0
G1_Anemia_before	0.26	[0.030-1.9]	0.19
G1_Neutropenia_before	0.11	[0.010-0.84]	**0.034**
G1_Lymphopenia_before	0.10	[0.030-0.32]	**< 0.001**
G1_Monocytopenia_before	0.08	[0.010-0.59]	**0.013**
G2_Altered_GFR_before	0.28	[0.060-1.2]	0.088
G1_ALT_increased_before	1.9	[0.25-14]	0.54

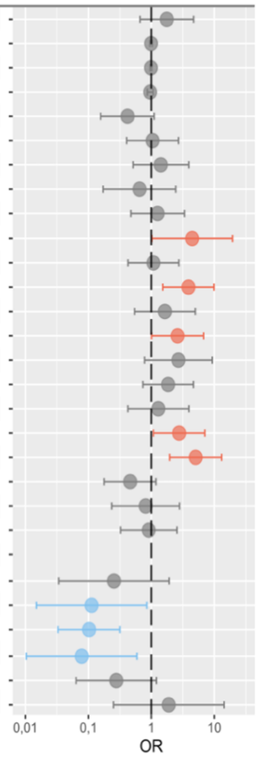

OR: Odds ratio; 95%CI: 95% Confidence interval; P: Wald test P-value; G: Grade; BMI: Body mass index; NEN: Neuroendocrine neoplasm; LysArg: Lysine-arginine solution; GFR: Glomerular filtration rate; ALT: Alanine aminotransferase
